# Opioids and the kidney: two sides of the same coin

**DOI:** 10.3389/fphar.2024.1421248

**Published:** 2024-07-25

**Authors:** Shaowei Gao, Qiulan He

**Affiliations:** Department of Anesthesiology, The First Affiliated Hospital of Sun Yat-sen University, Guangzhou, China

**Keywords:** opioid receptor, kidney, ischemia-reperfusion injury, renal function, acute renal failure, chronic kidney disease

## Abstract

Renal dysfunction, including acute renal failure (ARF) and chronic kidney disease (CKD), continues to present significant health challenges, with renal ischemia-reperfusion injury (IRI) being a pivotal factor in their development and progression. This condition, notably impacting kidney transplantation outcomes, underscores the urgent need for innovative therapeutic interventions. The role of opioid agonists in this context, however, remains a subject of considerable debate. Current reviews tend to offer limited perspectives, focusing predominantly on either the protective or detrimental effects of opioids in isolation. Our review addresses this gap through a thorough and comprehensive evaluation of the existing literature, providing a balanced examination of the dualistic nature of opioids’ influence on renal health. We delve into both the nephroprotective and nephrotoxic aspects of opioids, dissecting the complex interactions and paradoxical effects that embody the “two sides of the same coin” phenomenon. This comprehensive analysis is vital for understanding the intricate roles of opioids in renal pathophysiology, potentially informing the development of novel therapeutic strategies for preventing or treating hypoxic kidney injury.

## Highlights

1. Current research shows that while some opioids may help prevent damage from reduced blood flow and restoration to the kidneys, others may lead to negative effects such as increased cell death or disruption of kidney function.
2. Opioids may have a complex and dual role in kidney health, where they can both protect and harm the kidneys, depending on various factors such as dosage and specific opioid receptors targeted.
3. There is a need for more detailed and standardized research to fully understand how different opioids affect kidney health, which could lead to better and safer treatments for kidney-related diseases.

## 1 Introduction

Renal dysfunction, encompassing both acute renal failure (ARF) and chronic kidney disease (CKD), continues to be a significant health burden, with morbidity and mortality rates that have shown little improvement over the past 3 decades (Star, 1998; Imai et al., 2009). A pivotal factor in the development and progression of both acute and chronic renal dysfunction is renal ischemia-reperfusion injury (IRI), which is recognized as a leading cause and a common pathological pathway in these conditions (Liu et al., 2018).

Kidney transplantation remains the definitive treatment for end-stage renal disease, offering the best outcomes in terms of quality of life and longevity. However, the success of transplantation is frequently compromised by IRI, which adversely affects both short-term and long-term graft survival (Chatterjee, 2007; Wu et al., 2007). This highlights a critical gap in current medical practice, as effective strategies to mitigate renal IRI are notably lacking. The inadequacy of existing approaches underscores the urgent need for innovative therapeutic interventions to address this formidable challenge in renal transplantation (Wever et al., 2013).

Opioid receptors (ORs) and their ligands—encompassing both endogenous opioid peptides and exogenous opiate alkaloids—are known to affect a myriad of physiological functions, including sensory processes, neuroendocrine regulation, food consumption, and autonomic control (Mutoh et al., 2013). The identification of three classical OR subtypes—mu (MOR), delta (DOR), and kappa (KOR)—along with the less well-characterized nociceptin/orphanin FQ peptide (NOP; also referred to as opioid receptor-like 1 peptide) receptor, has significantly advanced our understanding of opioid pharmacology over recent decades (Mansour et al., 1987; Kapusta et al., 2005). Endogenous opioid peptides (EOPs), which are naturally occurring peptides within the body, include enkephalins, endorphins, and dynorphins. These peptides specifically bind to DOR, MOR, and KOR, and play a crucial role in modulating these physiological functions. For instance, enkephalins primarily interact with delta receptors, while beta-endorphins have high affinity for both mu and delta receptors, and dynorphins are more selective for kappa receptors. In contrast, exogenous opiate alkaloids, such as morphine, codeine, and oxycodone, are external substances introduced into the body. These alkaloids bind to ORs and can affect a broad range of physiological functions (Cox, 1982; Lefebvre, 1986). The widespread use of opioid agonists for pain management, particularly in the perioperative period of procedures such as kidney transplantation, and in treating pain associated with end-stage renal disease (Franco-Acevedo et al., 2020), underscores the importance of elucidating their effects on renal pathophysiology. However, this aspect remains a subject of considerable debate within current scientific literature. Some studies suggest a protective role of opioids against renal IRI (Habibey et al., 2010; Luo et al., 2020), akin to their reported benefits in other organs such as the heart and brain (He et al., 2013; Maslov et al., 2016). In contrast, other evidence indicates that activation of ORs may be detrimental to kidney health (Yoo et al., 2018; Dasari et al., 2021). This apparent contradiction may be attributed to the complex interplay of OR activation and the subsequent biological responses. For instance, opioid-induced sympathetic nerve excitation can contribute to AKI in the context of IRI (Mutoh et al., 2013), while, on the other hand, opioids might provide protective effects by mimicking ischemic preconditioning to alleviate renal IRI (Lee and Emala, 2001).

Existing literature reviews addressing the impact of opioids on renal diseases often present a limited perspective, tending to focus on either the protective or detrimental effects in isolation (Mercadante and Arcuri, 2004; Palomino et al., 2019; Mallappallil et al., 2021). In an effort to bridge this gap, our review aims to deliver a more balanced and comprehensive examination of the dualistic nature of opioids’ influence on renal health. We will delve into both the nephroprotective and nephrotoxic aspects of opioids, endeavoring to unravel the paradoxical “two sides of the same coin” phenomenon, drawing upon a wealth of literature evidence accumulated over recent decades. By dissecting these complex interactions, this review aspires to shed light on potential pathways that could inform the development of novel therapeutic strategies, aimed at either preventing or treating hypoxic kidney injury.

## 2 Expression of ORs in kidney

Understanding the localization of ORs is fundamental to comprehending how they exert their effects on the kidney. Historically, these receptors were primarily associated with the central nervous system (CNS), recognized for their role in mediating anti-nociception (Millan, 1986). Subsequent research expanded this view, revealing the presence of ORs in peripheral tissues, including the kidneys (Peng et al., 2012; Weber et al., 2013).

While the existence of ORs in renal tissues is now well-established, the exact distribution and prevalence of their subtypes continue to be debated. Table 1 collates findings from various studies that have explored the expression of ORs in the kidney across different species. Despite the diversity of methodologies and species studied, and the resulting discrepancies in outcomes, there is a general agreement on the consistent expression of KOR in the kidneys.

**TABLE 1:** The expression of opioid receptors (ORs) in the kidney across different species.

Research	MOR	DOR	KOR	Species	Specific tissue	Technique
Peng et al. (2012)	(−)	(++)	(+)	Human	ND	PCR
Weber et al. (2013)	(+)	(−)	(+)	Mouse	Mouse mesangial cells and whole kidney	PCR and Immunofluorescent staining
Quirion et al. (1983)	ND	ND	(+)	Rat	Kidney cortices	Radioligand Binding Assay
Slizgi and Ludens, (1985)	ND	ND	(+)	Rat	Whole kidney	Radioligand Binding Assay
Cai et al. (2016)	ND	ND	(+)	Mouse	Kidney cortex and medulla	KOR-Cre mediated recombination
Schmitt et al. (2017)	ND	ND	(+)	Mouse	Whole kidney	PET
Wittert et al. (1996)	(++)	(+)	(+)	Rat	Whole kidney	PCR
Lan et al. (2013)	(+)	(−)	(+)	Human and mouse	Podocytes	PCR and Immunofluorescent staining

Expression levels are indicated as: (−) for no expression, (+) for expression, and (++) for high expression, based on results reported in the literature. Abbreviations: ND, not determined; MOR, DOR, KOR, represent mu, delta, and kappa OR, respectively; PCR, polymerase chain reaction; PET, positron emission tomography.

Variations in the reported expression levels of MOR and DOR are not unexpected, considering the differences in experimental animal species, techniques used, and experimental conditions. To converge on a more coherent understanding of OR distribution in the kidney, future research endeavors would benefit from a concerted effort to standardize experimental protocols and methodologies across varied animal models. This approach would facilitate a more consistent and reliable interpretation of OR expression and their subsequent impact on renal physiology.

## 3 Functions of opioids on the kidney

The functional impact of ORs on renal physiology and pathology presents a more intricate and debatable landscape than the mere presence of these receptors in kidney tissue. In the ensuing sections, this review will undertake a comprehensive exploration of various common opioids along with specific agonists for each recognized subtype of ORs. The aim is to systematically dissect their individual as well as synergistic influences on renal function and pathology. This analysis is critical for unraveling the nuanced roles that these receptors and their ligands play in renal health and disease, thereby contributing to a more profound understanding of renal pharmacology and therapeutics.

### 3.1 Morphine

Morphine is recognized primarily as a potent MOR agonist (Ricarte et al., 2021); however, it also exhibits binding affinity to KOR and DOR (Chen et al., 1993). Although morphine stands as the most extensively studied opioid agonist within the corpus of opioid-related research, there remains a considerable lacuna in the literature regarding the specific OR subtypes implicated in its pharmacodynamic effects. The prevailing investigations into morphine’s action do not consistently delineate the particular receptor subtype—MOR, KOR, or DOR—to which its therapeutic and adverse effects can be attributed. Yet, a significant gap persists in discerning the effects attributable to specific OR subtypes. Historically, the scientific exploration into morphine’s effects initially prioritized understanding its influence on renal physiology. Kapusta et al. (1991) discerned a marked diminution in both urinary flow rate and sodium excretion subsequent to morphine administration in anesthetized rodents. On this basis, Flores et al. posited a dose- and temporally contingent paradigm, wherein lower morphine dosages may induce a transient augmentation of urine output within the initial hour following administration (Flores et al., 1997).

Pathological implications of morphine on renal health are subject to considerable debate. A number of investigations have postulated detrimental effects on the healthy kidney (Singhal et al., 1997; Atici et al., 2005; Weber et al., 2008; Lan et al., 2013; Luo et al., 2013; Jalili et al., 2017), with mechanisms encompassing apoptosis induction via mitochondria-dependent pathways (Luo et al., 2013), perturbation of the glomerular filtration apparatus (Lan et al., 2013), incitement of glomerulopathy (Weber et al., 2008), and facilitation of mesangial and medullary interstitial cell proliferation (Singhal et al., 1997; Weber et al., 2008). Notably, Weber et al. and Lan et al. have implicated KOR in mediating these harmful outcomes, rather than MOR, thus challenging conventional receptor-associated paradigms (Singhal et al., 1997; Lan et al., 2013).

Conversely, other scholarly works advocate for a renoprotective role of morphine in mitigating renal IRI (Habibey and Pazoki-Toroudi, 2008; Habibey et al., 2010; Pazoki-Toroudi et al., 2010; Bellini et al., 2016; Franco-Acevedo et al., 2020), with proposed mechanisms including the facilitation of vascular repair and angiogenic responses (Franco-Acevedo et al., 2020), enhancement of cell viability coupled with caspase modulation (Bellini et al., 2016), and the upregulation of nitric oxide production alongside inducible nitric oxide synthase expression (Habibey et al., 2010). The majority of these studies are rooted in animal experimentation, with a select few employing cellular models (Lan et al., 2013; Bellini et al., 2016).

Despite the dichotomous conclusions drawn by Bellini et al. and Lan et al., both research endeavors intriguingly implicate KOR as the pivotal site of morphine’s action within opossum kidney proximal tubular cells and human podocytes, respectively (Lan et al., 2013; Bellini et al., 2016). This denotes a compelling avenue for further inquiry, as the elucidation of receptor-specific pathways may afford critical insights into the nuanced roles of opioids in renal pathophysiology.

### 3.2 Fentanyl and its analogues

Fentanyl and its analogues represent a class of potent MOR agonists, exhibiting a higher affinity for MOR compared to morphine. Frequently, fentanyl is examined in conjunction with morphine due to their analogous effects on physiological processes, albeit with varying potencies (Bellini et al., 2016; Franco-Acevedo et al., 2020). Notably, remifentanil, an ultra-short-acting analogue of fentanyl, has garnered increasing attention in recent scientific inquiries. Xiong et al. demonstrated that pre-treatment with remifentanil ameliorates renal IRI in rodent models (Xiong et al., 2012). Corroborating these findings, Vianna et al. reported protective outcomes following the administration of remifentanil in isoflurane-anesthetized rats (Vianna et al., 2009). These observations from animal models received clinical substantiation through a retrospective study by Terashi et al., 2013, which indicated a temporary amelioration of renal function in perioperative adult CKD patients during orthopedic procedures.

Despite these insights, the consensus on the renoprotective effects of remifentanil remains tentative, with a pressing need for further empirical validation through meticulously structured experiments and clinical trials. The exploration into other fentanyl analogues, such as 4-fluoro-isobutyrylfentanyl, 4-chloroisobutyrylfentanyl, and isobutyrylfentanyl, is comparatively sparse. Ono et al. (2023) have delineated the nephrotoxic potential of these substances, positing respiratory depression mediated by MOP receptors as the underlying mechanism of renal impairment.

### 3.3 Tramadol

Tramadol, a less potent opioid, demonstrates a modest affinity for the MOR and weaker interactions with DOR and KOR (Raffa et al., 1992). The impact of tramadol on renal function is currently a subject of extensive debate. Sen et al. and Oliveira et al. observed that short-term administration of tramadol can decrease reactive oxygen species, thereby alleviating oxidative stress in animal models, suggesting a protective effect on the kidneys (de Oliveira et al., 2017; Sen et al., 2020). Contrarily, studies by Kizilkaya et al., 2002; Buhari et al., 2012 reported that tramadol’s impact on renal health in various animal models was neither beneficial nor harmful, indicating a neutral effect.

Further complicating the understanding of tramadol’s renal implications, Abdullah et al. and Elnagar et al. presented findings that suggest potential renal damage following prolonged use (exceeding 1 month) (Abdullah, 2018; Elnagar et al., 2022). This perspective is echoed in several clinical studies examining long-term opioid prescriptions, including tramadol, which have indicated potential harm to both healthy and transplanted kidneys during extended use (Barbosa-Leiker et al., 2016; Abbott et al., 2018; Novick et al., 2019). Although there is comparatively substantial evidence suggesting that long-term tramadol usage could be detrimental to kidney health, the implications of short-term use remain less clear and warrant further investigation.

### 3.4 Agonists with high selectivity of classical ORs

To delineate the distinct functionalities of various OR subtypes, researchers have employed highly selective agonists. This approach facilitates a more precise understanding of receptor-specific actions, particularly in renal physiology. A notable focus has been placed on KORs, with U-50488H, Asimadoline, and Niravoline serving as prototypical selective KOR agonists. Investigations utilizing these compounds have yielded consensus findings on the renal effects of KOR activation. Predominantly, activation of KOR has been associated with diuresis, antinatriuresis, and an increase in renal sympathetic nerve activity (Ashton et al., 1990; Kapusta and Obih, 1993; Moreau et al., 1996; Kramer et al., 2000; Gottlieb et al., 2005). Liu et al. (2018) conducted a notable animal study that observed a protective effect against renal IRI, purportedly mediated via the phosphatidylinositol 3 kinase (PI3K)/protein kinase B (AKT) signaling pathway. However, the necessity for additional research to corroborate these findings remains paramount.

In contrast, the understanding of MOR subtype functions has been informed by studies using highly selective MOR agonists, such as Dermorphin, cyclo [N-epsilon,N-beta-carbonyl-D-Lys (2) Dap (5)]enkephalinamide (cUENK6), and TAPP (H-Tyr-d-Ala-Phe-Phe-NH_2_). These studies consistently report diuretic effects following MOR activation (Kapusta et al., 1993; Gutkowska and Schiller, 1996; Gutkowska et al., 2004). However, there exists a notable divergence in findings related to sodium excretion. This discrepancy underscores the complexity inherent in MOR-mediated renal responses and highlights the need for further investigative efforts to elucidate these mechanisms more clearly.

Turning to the DOR, research has employed highly selective agonists such as UFP-512 (H-Dmt-Tic-NH-CH(CH2-COOH)-Bid) and DADLE ([D-Ala2, D-Leu5]-Enkephalin) to probe its renal implications. These studies have consistently observed protective effects on renal function, both in rat kidney epithelial cell models and in rat models of kidney disease (Habibey et al., 2010; Lian-Jin et al., 2010; Luo et al., 2020). While these observations regarding DOR agonists suggest potential avenues for therapeutic applications in renal pathologies, it is important to approach these conclusions with caution. The current body of research, although promising, requires further validation to ascertain the robustness and applicability of these findings. This is particularly crucial given the varying quality and scope of studies in this domain, which may impact the reliability of these preliminary conclusions.

Despite these advancements, a significant limitation of current research is the predominant reliance on animal models. This raises the question of how accurately these findings can be extrapolated to human physiology. Moreover, there exists a need to differentiate the effects mediated by central OR from those associated with receptors located directly in the kidney. While most studies have tended to overlook this aspect, recognizing and addressing this distinction is crucial for developing a more nuanced and comprehensive understanding of OR functions in renal physiology.

### 3.5 EOPs

EOPs are a fascinating class of molecules that have been found to influence various physiological systems in the body, including the kidneys. However, the literature on EOPs is relatively sparse, as very few studies directly use endogenous peptides to investigate opioid receptors, especially in the field of kidney research. This is largely due to the availability and convenience of using exogenous opioids in experiments. Despite this limitation, there is still some evidence that demonstrates the role of EOPs in renal function.

One of the key roles of EOPs in renal function is their contribution to renal adaptation to sodium restriction (Dibona and Jones, 1994). In normotensive rats, the inhibition of endogenous peripheral opioid mechanisms impaired the normal renal adaptive response to dietary sodium restriction. This suggests that EOPs are important for helping the kidneys adjust to changes in sodium intake.

EOPs also play a role in the regulation of renal responses during stress (Kapusta et al., 1989). Studies have shown that EOPs contribute to the antinatriuretic response (reduced sodium excretion) to environmental stress, which is mediated through the central nervous system. This indicates that EOPs are involved in helping the body conserve sodium during times of stress.

Another interesting effect of EOPs on renal function is their ability to stimulate renal growth (Haddox and Russell, 1979). The opioid peptide β-endorphin has been shown to increase the activity of renal ornithine decarboxylase, a marker of tissue growth response. This suggests that EOPs may be involved in regulating the growth and development of the kidneys.

Furthermore, Recent studies have identified proenkephalin (PENK), a stable surrogate marker for endogenous enkephalins, as a promising biomarker for kidney function. PENK is freely filtered by the glomerulus and has been shown to strongly correlate with glomerular filtration rate (GFR) (Ng et al., 2017; Schulz et al., 2017; Khorashadi et al., 2020). Moreover, elevated PENK concentrations have been linked to both acute kidney injury (AKI) and chronic kidney disease (CKD), as well as long-term kidney outcomes and mortality in various clinical settings, such as heart failure and acute myocardial infarction (Ng et al., 2014; Arbit et al., 2016; Matsue et al., 2017).

While the current evidence provides valuable insights into the role of EOPs, particularly enkephalins, in renal function, the specific mechanisms by which endogenous opioid peptides act on the kidneys remain unclear. The growing body of research on PENK as a novel biomarker for kidney function and its association with clinical outcomes highlights the potential importance of the opioid system in renal physiology. However, more research is needed to elucidate the precise pathways and signaling cascades involved in EOP-mediated regulation of renal function. Future studies should focus on using endogenous opioid peptides to investigate their effects on the kidneys and to unravel the complex interplay between the opioid system and renal physiology.

### 3.6 NOP

NOP, a 17-amino acid opioid-like peptide, is identified as the endogenous ligand for the NOP receptor, also known as opioid receptor-like 1 (Meunier et al., 1995). Although primarily located in the CNS, its presence in the kidney is less understood, with few studies indicating renal expression (Osinski et al., 1999). The role of NOP in renal physiology, particularly its diuretic and antinatriuretic effects, has been notably explored by Prof. Kapusta (Kapusta et al., 1997; 1999). These findings imply that NOP’s primary renal effects may be exerted indirectly, possibly outside the kidney.

The mechanisms by which the NOP receptor influences renal function, specifically in water and sodium balance, remain largely speculative. Calo et al. (2000) have suggested that these effects could be mediated through the inhibition of vasopressin secretion from supraoptic nucleus neurons. Research specifically addressing the impact of the NOP receptor on renal pathology is exceedingly scarce. Given the potential implications of NOP receptor modulation in renal function and its pathological states, there is a pressing need for further research in this area. Studies exploring both the molecular mechanisms of NOP receptor activity and its physiological effects on the kidney are essential to fully understand the role of this receptor system in renal health and disease.

### 3.7 Other opioids

The landscape of research on the renal effects of opioids beyond the commonly studied agonists is comparatively sparse. Buprenorphine and Biphalin, characterized by their broad affinity across all OR subtypes (Lukowiak et al., 2009; Davis et al., 2018), are two of the few examples in this field that have been studied. In a study conducted by Deng et al., 2000, it was observed that short-term administration of Buprenorphine did not significantly alter renal function in rat models. This finding suggests a potential renal safety profile for Buprenorphine, at least in the context of acute administration. Biphalin, a synthetic opioid with non-addictive properties, has also been the subject of renal studies. Notably, one study reported that Biphalin administration could maintain renal blood flow and reduce mean arterial pressure in spontaneously hypertensive rats, indicating potential therapeutic implications in hypertensive renal pathologies (Bądzyńska et al., 2016).

Naloxone, a non-selective opioid antagonist, has been observed in several studies to potentially benefit diseased kidneys in rat models (Mutoh et al., 2013; Takhtfooladi et al., 2016). The proposed mechanism for this beneficial effect might involve the inhibition of renal sympathoexcitation (Mutoh et al., 2013). Furthermore, the selective delta opioid antagonist 7-benzylindanylnaltrexone has shown promise in improving renal function in kidney-transplanted rats (Linner et al., 1998).

### 3.8 Sex differences in opioid effects on renal health

Despite the growing body of literature on the renal effects of opioids, there is a paucity of research specifically addressing sex differences in this context. While studies have demonstrated that sex can influence the analgesic effects of opioids, with some of these differences attributed to variations in pharmacokinetics and hormone levels between males and females (Craft, 2003; Fillingim and Gear, 2004), the potential sex-specific impact of opioids on renal function remains largely unexplored.

Animal studies have yielded inconsistent findings regarding sex differences in opioid-induced antinociception, with some reports suggesting greater sensitivity in males and others indicating no significant differences or even enhanced effects in females, depending on the specific opioid, dose, and pain model employed (Bartok and Craft, 1997; Craft, 2003). These discrepancies underscore the complexity of sex-related factors in modulating opioid responses and highlight the need for a more comprehensive examination of these differences across various experimental paradigms.

In the clinical setting, a systematic review and meta-analysis by Pisanu et al., 2019 found that while men and women may differ in their response to opioids for pain relief, these differences are significantly influenced by factors such as age and comorbid mental disorders. However, the role of these factors is not routinely considered in the prescription of opioids for pain management. The authors emphasize the urgent need for clinical trials that provide sex-specific data and account for potential confounding variables.

Given the established sex differences in opioid analgesia, which are partially mediated by pharmacokinetic factors and hormone levels (Craft, 2003; Huhn et al., 2018), it is plausible to hypothesize that sex may also play a role in the way opioids influence renal health. However, the current body of evidence is insufficient to draw definitive conclusions about these potential sex-specific effects. Future research should focus on elucidating the sex-based differences in the impact of opioids on renal function, with studies designed to investigate the pharmacokinetic and pharmacodynamic properties of opioids in the context of renal health, and a specific emphasis on sex as a variable. Additionally, clinical studies examining the long-term effects of opioid use on renal outcomes in both males and females would provide valuable insights into any potential sex-specific risks or protective factors.

The divergent effects of these opioid agonists and antagonists on the kidney highlight the complexity of OR subtypes and their varied influences on renal physiology. This complexity is compounded by the fact that the direction of these effects does not appear to be uniform across different agents, thereby challenging the establishment of a consensus. In the subsequent sections, we endeavor to dissect the underlying causes of this variability and explore the differential roles of OR subtypes in renal function.

## 4 What makes the coin’s two sides?

### 4.1 Central or peripheral

**FIGURE 1:**
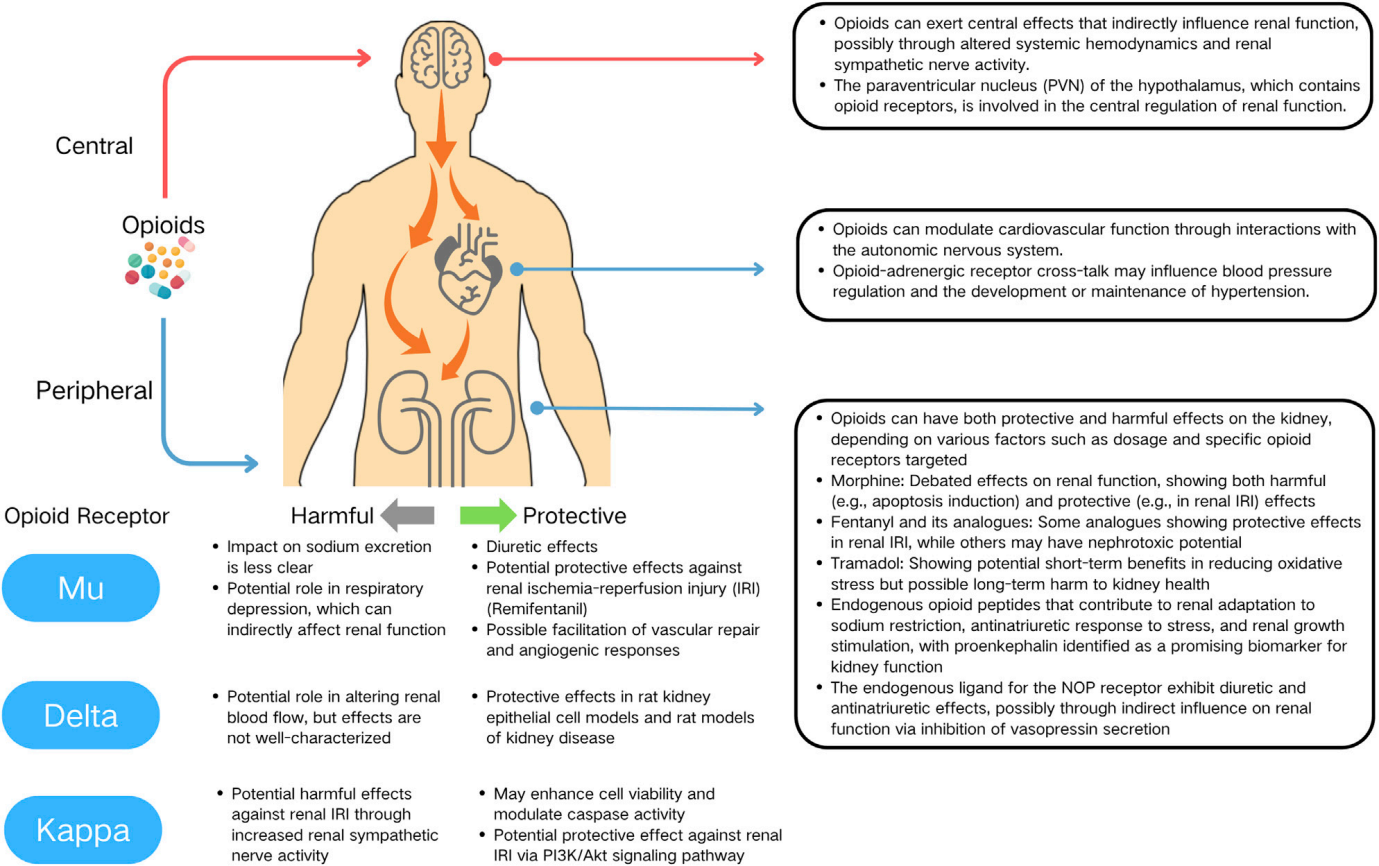
Multifaceted actions of opioids on the kidney, cardiovascular system, and central nervous system.

First, the pharmacokinetic properties of opioids, including their ability to cross the blood-brain barrier (BBB) and their distribution within different tissues, may play a significant role in modulating their renal effects. ORs, predominantly expressed in the CNS, play a pivotal role in mediating the systemic effects of most opioid drugs. A significant aspect of opioid pharmacodynamics is the ability of these drugs to traverse the BBB, thereby exerting central effects that can indirectly influence renal function. This interaction suggests that renal effects of opioids may not be solely attributed to their direct action on renal tissue but could also be a consequence of altered systemic hemodynamics and renal sympathetic nerve activity (RSNA) (Figure 1).

This hypothesis gains support from experimental findings by Prof. Kapusta and colleagues. They demonstrated divergent renal responses to opioids depending on the route of administration. Specifically, local administration of Dermorphine into the renal artery induced diuresis, whereas intracerebroventricular (icv.) administration resulted in antidiuresis (Kapusta et al., 1991; 1993). Further disparities were observed in experiments involving NOP receptor partial agonists. While icv. administration led to diuresis, intravenous administration did not produce a similar change in urinary flow (Kapusta et al., 2005). These results imply that the site of OR activation—central or peripheral—can distinctly modulate renal physiology.

#### 4.1.1 Where’s opioids’ central hub for kidney

Emerging evidence has begun to shed light on the specific brain regions that may play a crucial role in mediating the renal effects of opioids. The hypothalamus, particularly the paraventricular nucleus (PVN), has been identified as a critical region influencing RSNA (Katsurada et al., 2021). The PVN integrates neural and hormonal signals to regulate kidney function, with various neuropeptides and neurotransmitters modulating RSNA. Opioid receptors, such as KOR and MOR, are widely distributed in the hypothalamus, including the PVN (Gottlieb et al., 2005; Zhang et al., 2019). These receptors are involved in pain modulation and autonomic regulation, including sympathetic outflow (Zhang et al., 2019). The presence of KORs in the PVN raises the question of how opioids might regulate renal function through central mechanisms. A study by Gottlieb et al. (2005) provides valuable insights into this question. They demonstrated that microinjection of the KOR agonist U-50488H into specific subregions of the PVN produced differential cardiovascular and renal responses in anesthetized rats (Gottlieb et al., 2005). This finding suggests that KORs in the PVN are involved in the central regulation of renal function, possibly through modulation of RSNA. Undoubtedly, more detailed and in-depth research is eagerly needed to elucidate the brain regions and neural circuits involved in the central mechanisms by which opioids influence renal function. Future studies should focus on exploring the distribution of opioid receptors in the hypothalamus and other brain regions, their signaling pathways, and the regulatory mechanisms of renal sympathetic nerve activity.

#### 4.1.2 The crosstalk between OR and adrenergic receptors in the periphery

Opioids’ peripheral actions add further complexity through their interactions with other receptor systems, such as adrenergic receptors. Extensive evidence demonstrates functional interactions between the opioid and adrenergic receptor systems. This crosstalk involves second messenger systems, the formation of receptor heterodimers, and the presence of allosteric binding regions (Root-Bernstein and Churchill, 2021).

Several studies have shed light on the molecular mechanisms underlying opioid-adrenergic receptor interactions. For instance, Pepe et al. (1997) demonstrated that stimulation of δ-OR inhibits β1-adrenergic receptor (β1-AR)-induced positive inotropic effects and cAMP increases in rat heart (Pepe et al., 1997). This inhibitory crosstalk was found to be mediated by a pertussis toxin-sensitive G (i/o) protein involved in adenylyl cyclase inhibition. Similarly, Xiao et al. (1997) showed that the δ-OR agonist leucine-enkephalin markedly inhibited β1-AR-stimulated cAMP production and contractility in isolated rat heart (Xiao et al., 1997).

In the context of cardiovascular function and hypertension, Yu et al. (1999) investigated the crosstalk between cardiac κ-ORs and β-AR in developing hypertensive rats (Yu et al., 1999). They found that the inhibitory effect of κ-OR stimulation on β-adrenergic signaling was attenuated in the ventricular myocytes of 13-week-old spontaneously hypertensive rats with established hypertension, but not in younger rats before hypertension was fully developed, indicating that the blunted crosstalk between κ-ORs and β-ARs may contribute to the maintenance but not the initiation of hypertension in this model.

Given the presence of both opioid and adrenergic receptors in the kidney, it is plausible that similar interactions may occur in this organ, potentially influencing renal function and blood pressure regulation. The kidney plays a crucial role in maintaining fluid and electrolyte balance, as well as regulating blood pressure through various mechanisms, including the renin-angiotensin-aldosterone system and sympathetic nervous system. Opioid-adrenergic receptor crosstalk in the kidney may modulate these processes, thereby contributing to the development or maintenance of hypertension.

#### 4.1.3 Future directions

Despite these insights, there remains a gap in understanding how these differing receptor sites might influence the progression of renal diseases, such as IRI or renal functional failure. Most opioids, capable of freely crossing the BBB, could produce mixed and complex effects in physiological and pathological states, unlike the more controlled experimental conditions. Future advancements in this field may depend on the development of new pharmaceutical agents, designed to be impermeable to the BBB, such as Loperamide (Proskuryakova et al., 2009). Such drugs could offer more precise insights into the peripheral *versus* central actions of opioids on renal function, thereby enhancing our understanding of opioid-related renal pathophysiology.

### 4.2 Study design issues

Secondly, it is imperative to consider the impact of study design, particularly the distinction between clinical studies and animal experiments, on the outcomes of opioid-related renal research. Variations in experimental conditions, such as the choice of model organism and the duration of opioid exposure, are critical factors that likely contribute to the divergent findings observed in the literature.

Clinical studies, in particular, have tended to report deleterious effects of opioids on renal function (Barbosa-Leiker et al., 2016; Novick et al., 2016; Babak et al., 2017; Abbott et al., 2018; Fassio et al., 2018; Dasari et al., 2021). Notably, these studies often involve participants who have been exposed to opioids over extended periods. Furthermore, the opioids used in these clinical contexts are typically a combination of various types, rather than a single, specific agent. This polypharmacy aspect could potentially confound the attribution of renal harm to any one opioid compound, despite the well-established evidence that long-term use of traditional opioids is linked to adverse renal outcomes.

In light of these considerations, future research might beneficially focus on novel OR agonists that demonstrate minimal side effects and exhibit high affinity for specific receptor subtypes. An encouraging trend in this direction is evident in the research surrounding Remifentanil (Vianna et al., 2009; Xiong et al., 2012; Terashi et al., 2013). Studies on this agent have consistently reported protective effects on renal function, suggesting a potentially safer profile compared to traditional opioids. Such findings provide a compelling rationale for further exploration into selective OR agonists as a means to mitigate the renal risks associated with opioid therapy.

### 4.3 Heterogeneity

Finally, individual genetic variations among subjects, which can affect receptor expression and opioid metabolism, might also contribute to the variability in response to opioid agonists.

These considerations underscore the need for a more nuanced approach to studying opioid effects on the kidney, taking into account the multifaceted nature of opioid-receptor interactions and the diverse physiological contexts in which they operate.

## 5 Conclusion

In conclusion, this review highlights the multifaceted impact of OR agonists on renal function, illustrating a complex interplay of protective and harmful effects. It underscores the necessity for a deeper understanding of the central and peripheral actions of these agonists and calls for standardized research methodologies. Such insights are crucial for advancing therapeutic strategies in managing renal pathologies, ultimately contributing to better patient outcomes in renal health and disease management.
